# In Vivo Assessment of Deep Vascular Patterns in Murine Colitis Using Optoacoustic Mesoscopic Imaging

**DOI:** 10.1002/advs.202404618

**Published:** 2024-10-22

**Authors:** Adrian Buehler, Emma L. Brown, Emmanuel Nedoschill, Markus Eckstein, Petra Ludwig, Felix Wachter, Henriette Mandelbaum, Roman Raming, Mariam‐Eleni Oraiopoulou, Lars‐Philip Paulus, Ulrich Rother, Oliver Friedrich, Markus F. Neurath, Joachim Woelfle, Maximilian J. Waldner, Ferdinand Knieling, Sarah E. Bohndiek, Adrian P. Regensburger

**Affiliations:** ^1^ Department of Pediatrics and Adolescent Medicine University Hospital Erlangen Friedrich‐Alexander‐Universität (FAU) Erlangen‐Nürnberg 91054 Erlangen Germany; ^2^ Cancer Research UK Cambridge Institute University of Cambridge CB2 0RE Cambridge United Kingdom; ^3^ Institute of Pathology Friedrich‐Alexander‐Universität (FAU) Erlangen‐Nürnberg 91054 Erlangen Germany; ^4^ Department of Vascular Surgery University Hospital Erlangen Friedrich‐Alexander‐Universität (FAU) Erlangen‐Nürnberg 91054 Erlangen Germany; ^5^ Institute of Medical Biotechnology Department of Chemical and Biological Engineering Friedrich‐Alexander‐Universität (FAU) Erlangen‐Nürnberg 91052 Erlangen Germany; ^6^ Department of Medicine 1 University Hospital Erlangen Friedrich‐Alexander‐Universität (FAU) Erlangen‐Nürnberg 91052 Erlangen Germany

**Keywords:** dextran sodium sulfate induced colitis, inflammatory bowel disease, murine acute colitis, optoacoustic imaging, photoacoustic imaging, raster‐scanning optoacoustic mesoscopy, vessel morphology analysis

## Abstract

The analysis of vascular morphology and functionality enables the assessment of disease activity and therapeutic effects in various pathologies. Raster‐scanning optoacoustic mesoscopy (RSOM) is an imaging modality that enables the visualization of superficial vascular networks in vivo. In murine models of colitis, deep vascular networks in the colon wall can be visualized by transrectal absorber guide raster‐scanning optoacoustic mesoscopy (TAG‐RSOM). In order to accelerate the implementation of this technology in translational studies of inflammatory bowel disease, an image‐processing pipeline for TAG‐RSOM data has been developed. Using optoacoustic data from a murine model of chemically‐induced colitis, different image segmentation methods are compared for visualization and quantification of deep vascular patterns in terms of vascular network length and complexity, blood volume, and vessel diameter. The presented image‐processing pipeline for TAG‐RSOM enables label‐free in vivo assessment of changes in the vascular network in murine colitis with broad applications for inflammatory bowel disease research.

## Graphical abstract

Transrectal absorber guide raster‐scanning optoacoustic mesoscopy (TAG‐RSOM) enables transabdominal in vivo imaging of murine colitis. For advanced visualization and quantification of deep vascular patterns different image segmentation methods are applied. The presented image‐processing pipeline for TAG‐RSOM enables label‐free in vivo assessment of changes in the vascular network in murine colitis with broad applications for inflammatory bowel disease research.

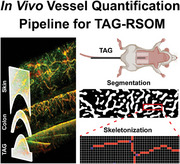

## 1 Introduction

The human intestine remains a difficult target for medical imaging modalities. While colonoscopy provides high resolution, it is limited to the mucosa. Tomographic procedures, aided by magnetic fields or X‐rays, visualize deep‐seated tissue, yet are limited by a resolution of 0.1–1 mm.^[^ ^1^ ^]^ Ultrasound (US) is easy to apply to assess the extent of inflammation but suffers from low contrast and low specificity.^[^ ^2^ ^]^

Optoacoustic (also called photoacoustic) imaging (OAI) is a molecular imaging technique that combines optical contrast with depth resolution,^[^ ^3^ ^]^ converting pulsed radiant energy into acoustic waves.^[^ ^4^ ^]^ The resulting acoustic waves are subject to lower scattering and refraction effects compared to all‐optical imaging, hence they can be detected at the tissue surface to enable the precise reconstruction of the distribution of optically active molecules deep in the tissue.^[^ ^5^ ^]^ OAI thus benefits both from the contrast provided by optical absorption in tissue and from the high spatial and temporal resolution of US‐based imaging. This optimal use of these respective underlying physical principles leads to a favorable trade‐off between penetration depth and resolution while decoding molecular information through absorption properties offers advantages over traditional ultrasound.

Absorption of light in tissue depends on the optical properties of endogenous chromophores and/or contrast agents present.^[^ ^6^ ^]^ Hemoglobin, featuring an iron ion as its coordination center, exhibits high absorption of visible and near‐infrared light and holds promising clinical applications for OAI, in cardiovascular,^[^ ^7^ ^]^ inflammatory,^[^ ^8^ ^]^ and various other diseases including dermatological pathologies and cancer.^[^ ^9^ ^]^ In studies using hand‐held multispectral optoacoustic tomography (MSOT), the disease burden of patients with Crohn's disease or ulcerative colitis can be monitored based on the hemoglobin content of the intestinal wall.^[^ ^10^ ^]^ In inflammatory bowel disease (IBD) research, vascular changes are of great interest, as vascular damage and the subsequent activation of the hyper thrombotic state have been shown to be promising targets for future therapeutic interventions.^[^ ^7^, ^11^ ^]^ OAI might fill the gap of imaging microvascular changes non‐invasively and in vivo.

Transrectal absorber guide raster‐scanning optoacoustic mesoscopy (TAG‐RSOM) is a new method for non‐invasive label‐free imaging of the murine intestine in high resolution (40 µm lateral and 10 µm axial) in vivo. The transrectal absorber guide (TAG) enables precise localization and visualization of the colon wall and intestinal vessels with a diameter down to 76.78 ± 7.96 µm in murine models of colitis over a field‐of‐view (FOV) of 12 × 12 × 3 mm.^[^ ^12^ ^]^ We previously demonstrated using TAG‐RSOM imaging that colon wall thickness and optoacoustic signal intensity are promising biomarkers for the assessment of severity of acute colitis in mice,^[^ ^12^ ^]^ supporting the notion that OAI might allow early detection of inflammation and might be used for monitoring treatment response.

Understanding vascular network topology and its evolution through pathophysiological states is vital for characterizing disease status for inflammatory conditions. Quantification of vascular networks using OAI remains challenging due to the sparsity of the vascular networks and the presence of complex image artifacts arising from different system geometries, as well as the pathology‐specific nature of optical imaging biomarkers which was not achieved in the intestine in vivo before.^[^ ^13^ ^]^ Therefore, in this work, we present an analysis pipeline for TAG‐RSOM data tailored to quantify vascular changes in the murine colon in terms of vessel length, diameter, blood volume, and network complexity among other descriptors, based on pre‐processing, segmentation, and quantification methods. Next, we demonstrated the potential of our analysis pipeline to monitor vascular changes in a model of mild and severe acute colitis to facilitate TAG‐RSOM as a novel instrument for pre‐clinical IBD research.

## 2 Results

**Figure 1:**
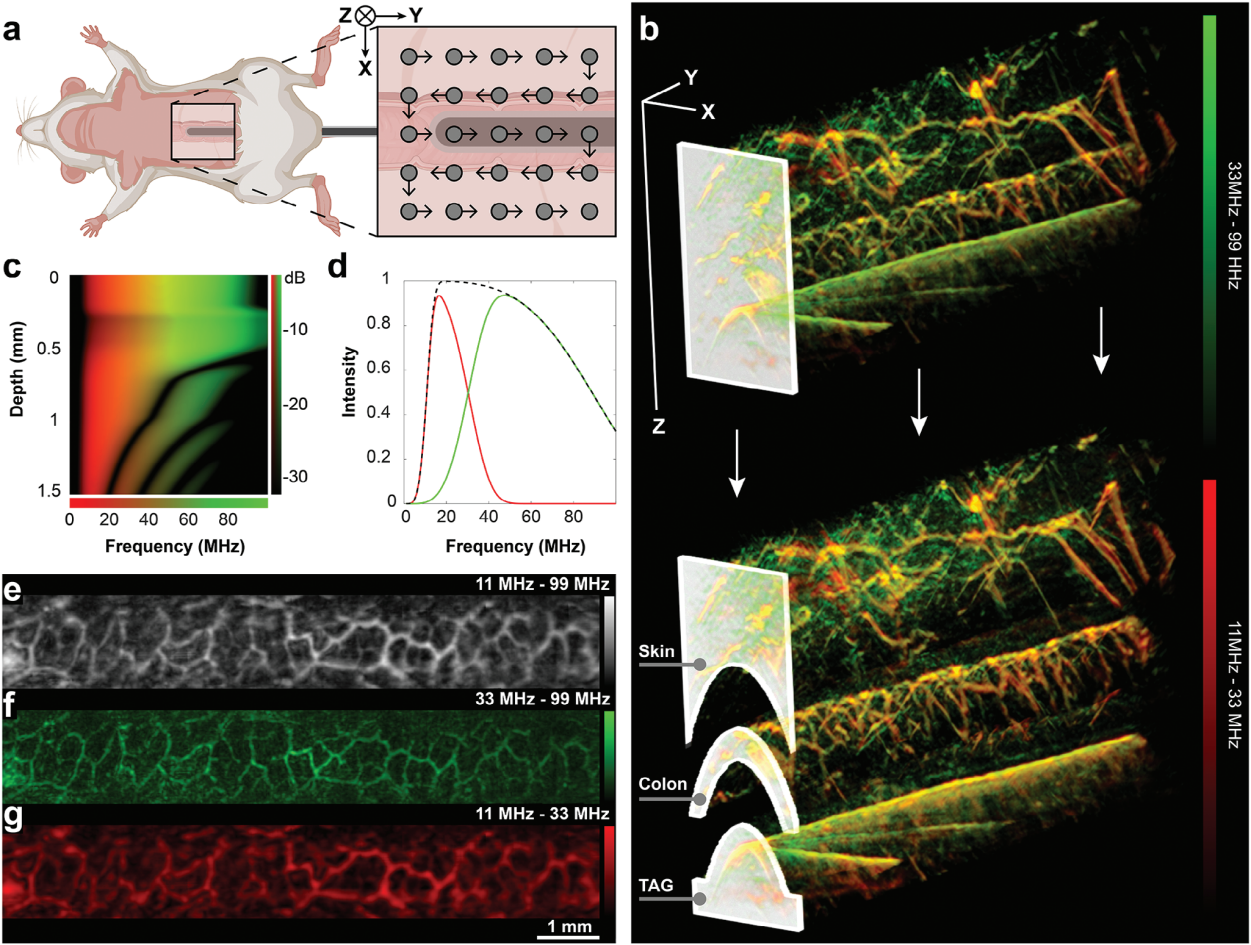
Transrectal absorber guide raster‐scanning optoacoustic mesoscopy. a) Schematic illustration of transrectal absorber guide (TAG) placement and the pattern used for raster‐scanning optoacoustic mesoscopy (RSOM). b) TAG‐RSOM facilitates placement of the colon into the imaging depth of the RSOM and the exact identification of the intestinal wall. Scale is 1 mm for each dimension. c) Simulated depth‐dependent frequency response of the employed RSOM, adapted from Schwarz et al.^[^ ^14^ ^]^ d) RSOM is capable of measuring frequencies between 11 and 99 MHz (dashed line). High (33–99 MHz, green line) and low pass filter (11–33 MHz, red line) were used to extract small and large structures, respectively. e) 2D average intensity projection of the colon shown in 3D in (b) shows the entire frequency band. When small structures and noise f) are removed, larger structures in deeper tissue can be measured g). Created with BioRender.com.

TAG‐RSOM offers a novel approach for pre‐clinical in vivo evaluation of the murine colon vasculature (**Figure** **1**a; Figure S1, Supporting Information). The TAG is formed from a thin glass capillary inserted within a sealed transparent polypropylene tube to enable fluid flow between the inner and outer tubes. The capillary provides the necessary rigidity to localize the colon wall close to the surface of the animal, while the polypropylene tube enables the exchange of fluid from a high‐contrast dye solution (visible within the RSOM images) to water, virtually transparent at the excitation wavelength used (Figure 1b).^[^ ^12^ ^]^

Acoustic waves generated during RSOM imaging experience frequency‐dependent attenuation. The RSOM system used in this study employs a single transducer with a center frequency of 50 MHz. Simulated human palm tissue was used by Schwarz et al. to generate system‐specific frequency response as a function of depth (Figure 1c).^[^ ^14^ ^]^ Therefore, to compensate for the decreasing detection bandwidth in deep tissue and provide the best stability of the vessel network analysis pipeline introduced in this work, a low‐pass filter (11–33 MHz) was applied before image reconstruction (Figure 1d–g).

The coupling of the RSOM scan head and the skin of the subject constitute opposing surfaces that cause periodic reflection artifacts. To mitigate these, a Gaussian filter with a 3D kernel and subsequent rolling ball background subtraction was employed.^[^ ^13^ ^]^ The TAG itself can represent a source of similar artifacts when filled with high‐contrast dye solution that are caused by the high signal intensity of the contrast, however, the TAG facilitates contrast changes without repositioning of the subject; analysis undertaken here is based on RSOM scans acquired when the TAG is filled with water.

Complex 3D imaging targets rely on sophisticated segmentation methods, requiring large amounts of high‐quality labeled data. Fortunately, colon imaging using TAG‐RSOM increases the cross section of the hollow organ and stretches the intestinal wall to a thin layer at constant depth (Figure S2, Supporting Information). In this state, the colon wall thickness is 60.90 ± 20.67 µm in healthy and 127.3 ± 53.65 µm in mice with severe colitis when measured with TAG‐RSOM.^[^ ^12^ ^]^ Considering that most venules and arterioles are located between submucosa and mucosa, the 10 µm axial resolution offered by the RSOM, and the limited view of 36° along the colon axis, analysis of the vascular network inside the intestinal wall was approximated here in two dimensions, with analysis performed on average intensity projections of the extracted colon wall. Furthermore, analysis in two dimensions eliminates the need for learning‐based segmentation methods and enables the use of transparent, rule‐based methods analogous to other 2D vessel segmentation applications.

**Figure 2:**
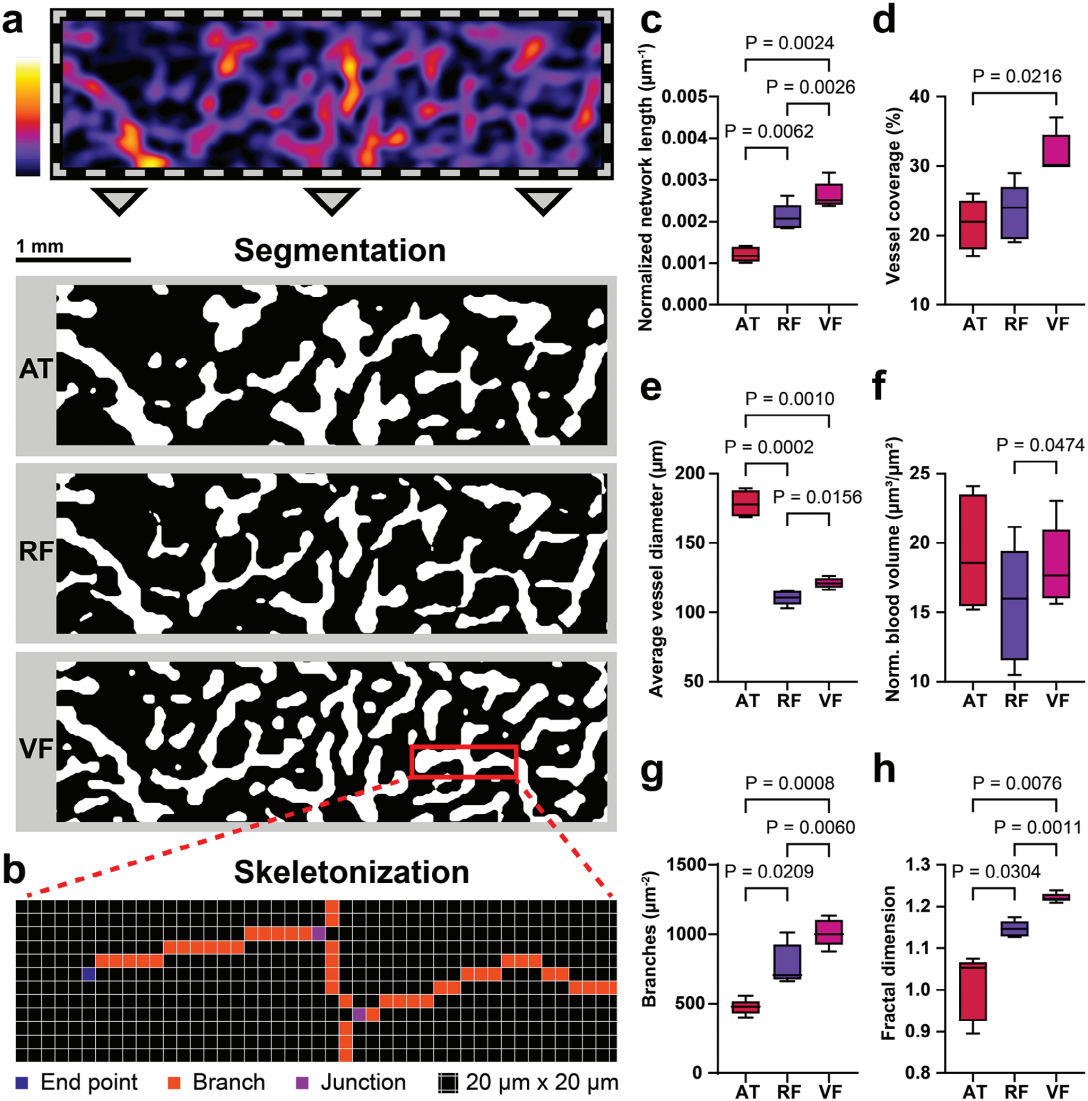
Image segmentation methods. a) The pre‐processed images (illustrated using an exemplary image section) were segmented using three different methods: Auto threshold (AT), trained Random Forest (RF), and Frangi vesselness filter (VF). b) The segmented images were skeletonized, and statistics were calculated based on the segmented area, number of end‐points, branches, and junctions. Normalized network length c), vessel coverage d), average vessel diameter e), normalized blood volume f), the number of branches g), and the fractal dimension h) were calculated based on the segmented (d) or skeletonized images (g,h), or a combined analysis (c,e,f).

One learning‐based and two rule‐based segmentation methods were employed to separate blood vessels from the background (**Figure** **2**a,b). The methods used were: auto threshold (AT), random forest classifier (RF), and Frangi vesselness filter^[^ ^15^ ^]^ in combination with thresholding (VF). The segmentation methods differed in their ability to detect blood vessels. Segmented images of healthy mouse colons from a sham group at day 0 were skeletonized and used as a benchmark to compare the methods. While AT solely relies on intensity features, resulting in the loss of smaller faint vessels, RF‐based segmentation was able to segment smaller structures, leading to a significant increase in vessel length when normalized to the analysis area (AT vs RF: 1.21 ± 0.18 µm^−1^ × 10^−3^ vs 2.11 ± 0.32 µm^−1^ × 10^−3^, P = 0.0062, Figure 2c). Interestingly, RF did not overestimate the width of bright structures, resulting in segmentation of a comparable vessel coverage (AT vs RF: 21.60 ± 3.65% vs 23.40 ± 4.04%, P > 0.9999, Figure 2d) but a significantly smaller average vessel diameter (AT vs RF: 178.5 ± 9.5 µm vs 110.6 ± 5.2 µm, P = 0.0002, Figure 2e). By comparison, VF segmented an even larger number of vessels, as reflected in a larger normalized network length (VF vs RF: 2.63 ± 0.32 µm^−1^ × 10^−3^ vs 2.11 ± 0.32 µm^−1^ × 10^−3^, P = 0.0026, Figure 2c) and larger vessel coverage (VF vs RF: 31.8 ± 3.03% vs 23.40 ± 4.04%, P = 0.1195, Figure 2d), while providing similar precision compared to RF. Subsequently, this observation is furthermore expressed by an increased number of branches (VF vs RF: 1012 ± 98.3 cm^−2^ vs 781.3 ± 146.6 cm^−2^, P = 0.0060, Figure 2g) and higher fractal dimension (VF vs RF: 1.22 ± 0.01 vs 1.15 ± 0.02, P = 0.0011, Figure 2h). Finally, the estimated normalized blood volume calculated based on AT and VF segmentations is comparable (AT vs VF: 19.29 ± 4.06 µm^3^ µm^−2^ vs 18.32 ± 2.91 µm^3^ µm^−2^, P = 0.8670, Figure 2f), in contrast to the normalized blood volume segmented by RF (VF vs RF: 18.32 ± 2.91 µm^3^ µm^−2^ vs 15.59 ± 4.21 µm^3^ µm^−2^, P = 0.0474, Figure 2f). In summary, VF and RF demonstrated equivalent segmentation results based on visual inspection, and both outperformed AT (Figure 2a). However, to mitigate potential bias resulting from a limited number of training data sets, the rule‐based VF was selected for all subsequent analyses over RF. Consequently, subsequent data analyses exclusively rely on VF. All corresponding calculations based on the AT and RF segmentation methods can be found in the supplement (Figures S3–S6, Supporting Information).

**Figure 3:**
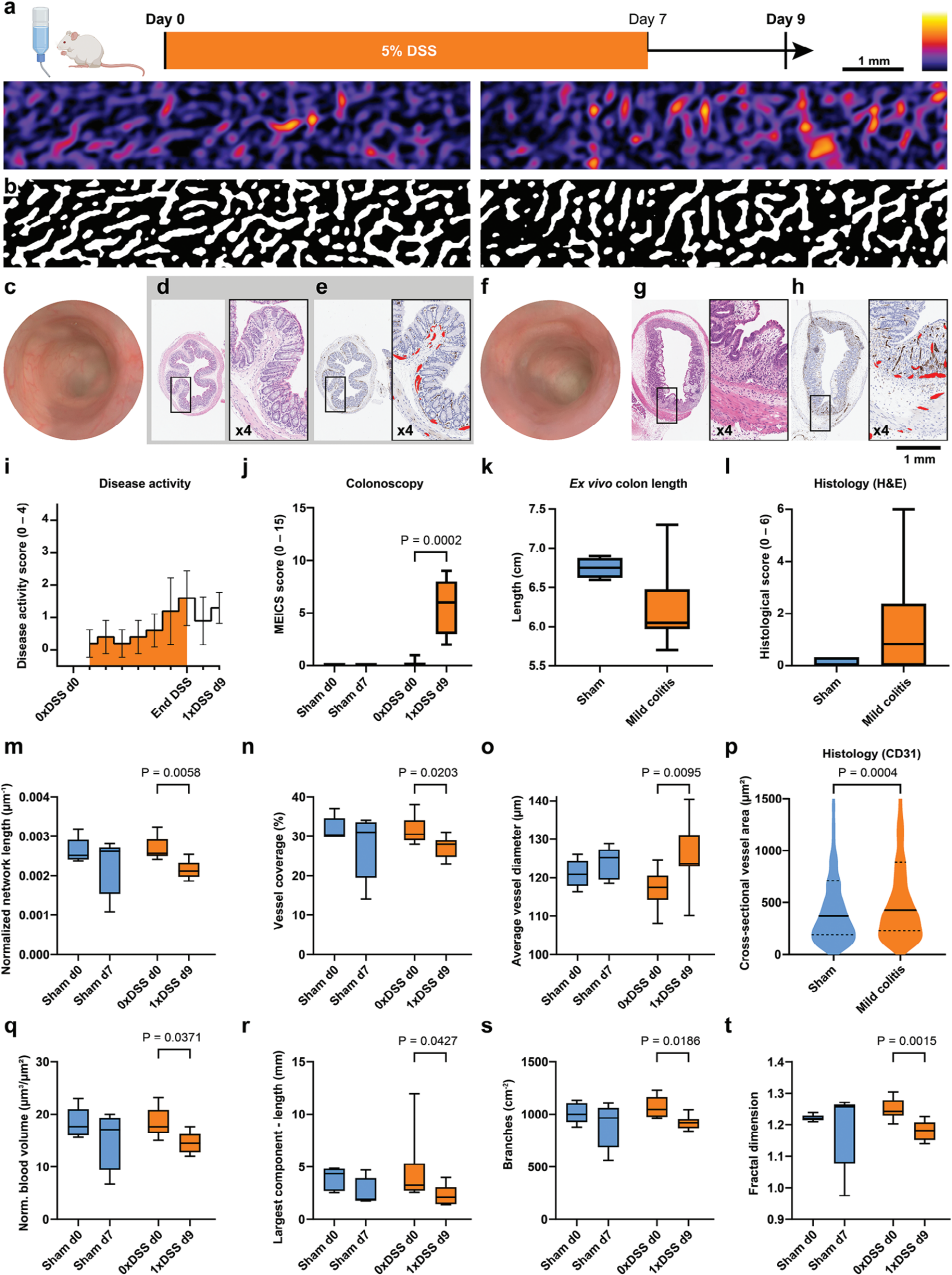
Quantification of vessel network evolution during mild colitis. a) Exemplary TAG‐RSOM images of the healthy colon of a mouse before one week of 5% DSS administration (day 0, left) and of the inflamed colon two days after DSS administration (day 9, mild colitis, right). Scale bar 1 mm. b) Segmentation of the images into vessel (white) and background (black) using Frangi vesselness filter with thresholding. Exemplary colonoscopy and histology data of sham c–e) compared to mild colitis f–h): Colonoscopy revealed a slightly increased granularity, mild changes in the vascular pattern and reduction of mucosal transparency in the diseased colon on day 9 (f) compared to the healthy colon at day 0 (c). H&E stained histological colon section of sham group mouse (d) shows no defects in contrast to partly missing epithelia and severely altered crypt architecture in the colon of a diseased mouse (g). CD31 staining reveals the endothelial cells of blood vessels and were used to manually outline the cross‐sectional vessel area (shown in 4x magnification in red) in the healthy (e) and the inflamed colon (h). The disease models were further validated by disease activity score i) (mean and standard deviation), colonoscopy j), ex vivo colon length measurements k), and histopathological scoring l) adopted from *Buehler* et al.^[^ ^12^ ^]^ m‐o) TAG‐RSOM parameters: Normalized network length, vessel coverage, and average vessel diameter. p) Cross‐sectional vessel area determined by CD31‐stained histological sections of sham (total number of vessels = 597) and mild colitis (total number of vessels = 1165). q–t) TAG‐RSOM parameters: Normalized blood volume, largest connected component in the network by length, normalized number of branches, and fractal dimension. Sham group N = 5 (blue), mild colitis N = 10 (orange). Whiskers correspond to minimum and maximum value.

Mild acute colitis was induced by oral administration of a 5% dextran sulfate sodium (DSS) solution for seven days (Figure S7, Supporting Information). Imaging was performed before DSS administration at day 0 (d0) to assess the healthy state and two days after treatment at day 9 (d9) in order to warrant clear inflammation (**Figure** **3**a,b; Figure S8, Supporting Information). Disease activity monitoring, colonoscopy, ex vivo colon length, and subsequent histology confirmed a mild inflammation (Figure 3c–l). Quantification of the vasculature network using the VF pipeline revealed changes regarding all descriptors used for quantification (**Table** **1**). The normalized vessel length (d0 vs d9: 2.68 ± 0.27 µm^−1^ × 10^−3^ vs 2.16 ± 0.24 µm^−1^ × 10^−3^, P = 0.0058, Figure 3m) and vessel coverage (d0 vs d9: 31.40 ± 3.13% vs 27.00 ± 2.58%, P = 0.0203, Figure 3n), both calculated relative to the analysis area, decreased significantly in the mild colitis group, while no significant effect was observed within the sham control group. As a result, the average vessel diameter increased during inflammation (d0 vs d9: 117.1 ± 4.7 µm vs 125.5 ± 7.8 µm, P = 0.0095, Figure 3o), which indicates a loss of smaller vessels and/or dilation of existing ones. These findings could reflect the increasing trend in average cross‐sectional vessel area measured ex vivo by histopathology (sham d7 vs mild colitis d9: 731.0 ± 2066 µm^2^ vs 822.1 ± 1228 µm^2^, P = 0.0004, Figure 3p and Figure S9, Supporting Information), although this was not significant.

**Table 1:** Network descriptors.

Network descriptor	Description
Normalized network length	The total length of the skeletonized segmented network divided by the analysis area
Vessel coverage	The total segmented area divided by the analysis area
Average vessel diameter	Segmented area divided by the total length of the network
Normalized blood volume	Sum of the volumes of cylinders with diameter according to the local vessel diameter normalized on the analysis area
Largest component by length	Length of the skeletonized largest connected component segmented
Normalized number of branches	A network branch is defined as a connection between two intersections, two end‐points or an intersection‐end‐point‐connection
Fractal dimension	The fractal dimension (scaling rule) is calculated based on the logarithmic regression of the relation between box size and count

A total of seven parameters were used to describe the vasculature in the colon in two dimensions. Details about image processing or descriptor calculations can be found in the repository https://github.com/AdrianBuehler/TAG‐RSOM‐Vessel‐Analysis.

Inflammatory processes in the colitis state were also accompanied by a significant decrease in blood volume (d0 vs d9: 18.33 ± 2.61 µm^3^ µm^−2^ vs 14.64 ± 1.90 µm^3^ µm^−2^, P = 0.0371, Figure 3q), which was not observed in the sham control group. Furthermore, the largest connected component in the network by length (d0 vs d9: 4.65 ± 3.33 mm vs 2.34 ± 0.93 mm, P = 0.0427, Figure 3r), and the number of branches of the network relative to the analysis area decreased (d0 vs d9: 1070 ± 98.0 cm^−2^ vs 917.6 ± 61.7 cm^−2^, P = 0.0186, Figure 3s). Finally, the vascular networks in the colon of mice with mild acute colitis exhibit a reduced complexity, which was quantified by a decreasing fractal dimension (d0 vs d9: 1.25 ± 0.03 vs 1.18 ± 0.03, P = 0.0015, Figure 3t).

**Figure 4:**
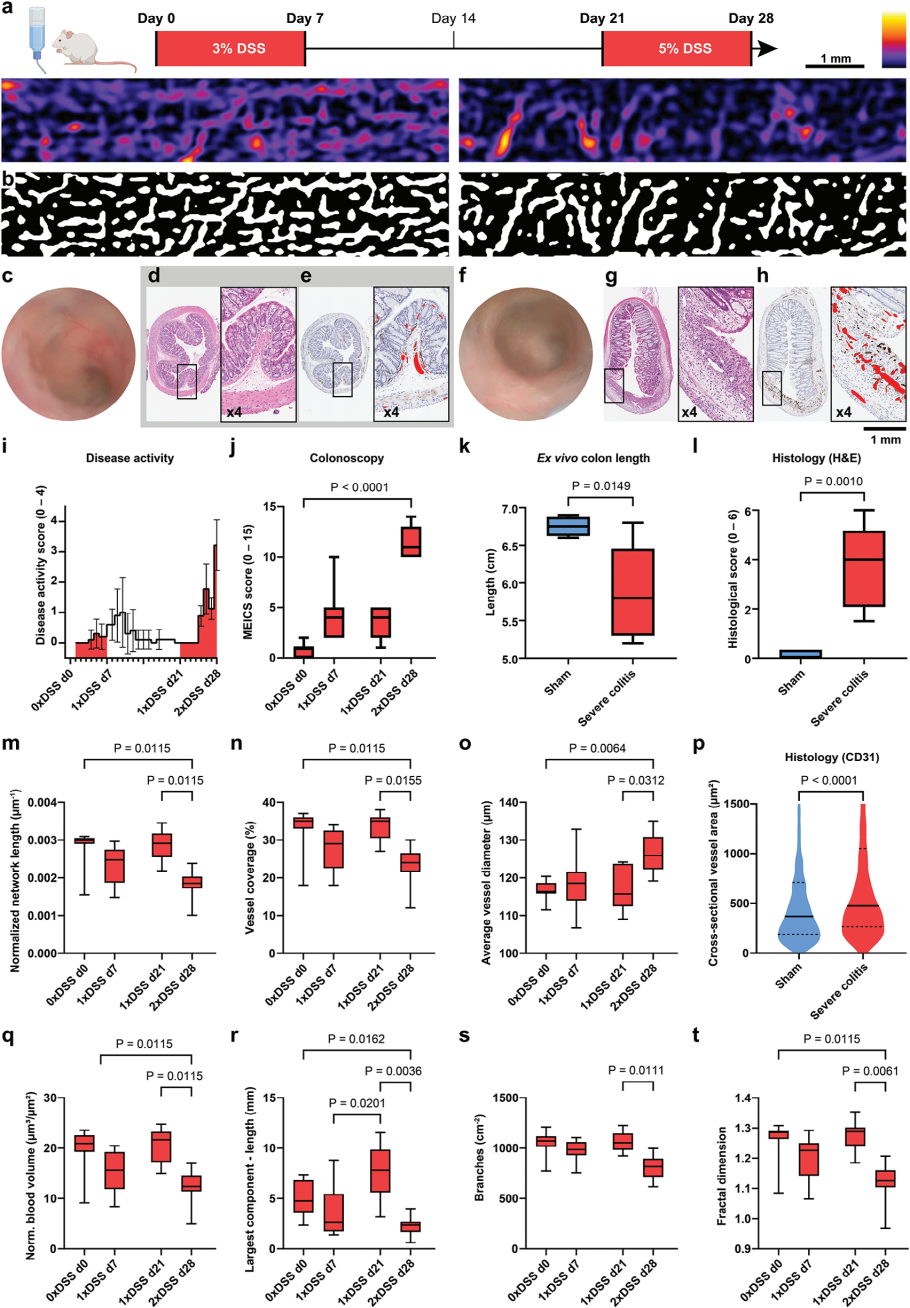
Quantification of vessel network evolution during severe colitis. a) Exemplary TAG‐RSOM images of a severely diseased mouse (right) appear faint with fewer small structures and patchy bigger vessels, when compared to the healthy state at day 0 (left). b) Frangi vesselness filter segments fewer vessels at day 28 (right), compared to the healthy state at day 0 (left). Exemplary colonoscopy and histology data of sham c–e) compared to mild colitis f–h): In contrast to the healthy colon at day 0 (c), an increased granularity and the non‐transparent colon wall transparency indicates a severe inflammation on day 28 (f). H&E stained histological sections of a healthy (d) and an inflamed colon (g). CD31 staining reveals the endothelial cells of blood vessels and were used to manually outline the cross‐sectional vessel area (shown in 4x magnification in red) in the healthy (e) and the inflamed colon (h). The disease models were further validated by disease activity score i) (mean and standard deviation), colonoscopy j), ex vivo colon length measurements k), and histopathological scoring l) as adopted from *Buehler* et al.^[^ ^12^ ^]^ m–o) TAG‐RSOM parameters: Normalized network length, vessel coverage, and average vessel diameter. p) Cross‐sectional vessel area determined by CD31 stained histological sections of sham (total number of vessels = 597) and severe colitis (total number of vessels = 984). q–t) TAG‐RSOM parameters: Normalized blood volume, largest connected component in the network by length, normalized number of branches, and fractal dimension. Sham group N = 5 (blue), severe colitis N = 9 (red). Whiskers correspond to minimum and maximum value.

Having examined vascular network changes during mild colitis, a severe colitis group was then studied, which underwent two cycles of DSS administration (**Figure** **4**). During the first cycle, mice received a 3% DSS solution for one week and developed mild inflammation. After a healing period of two weeks, a second cycle with an increased concentration of 5% DSS was administered to induce severe colitis (Figure 4a,b; Figure S7, Supporting Information). Imaging was performed before (d0 and d21) and on the last day (d7 and d28) of each cycle (Figure S10, Supporting Information). Mild inflammation after the first cycle (d7) and severe colitis after the second cycle (d28) were diagnosed by health scoring, weight monitoring, colonoscopy, as well as colon shortening, and histological scoring after the last imaging time point (Figure 4c–l).

After the first cycle of DSS administration, a slight decrease in normalized network length, vessel coverage, and resulting average vessel diameter was measured, indicating a mild inflammation as observed in Figure 3. At day 21, after two weeks of healing, the vascular network returned to an apparent healthy state without any overshooting reaction. All health parameters and measured network descriptors reverted to their baseline values. In contrast, a second cycle with higher DSS dose administration led to severe colitis as exhibited by significantly decreasing normalized network length (d21 vs d28: 2.85 ± 0.41 µm^−1^ × 10^−3^ vs 1.83 ± 0.37 µm^−1^ × 10^−3^, P = 0.0115, Figure 4m) and vessel coverage (d21 vs d28: 33.22 ± 3.60% vs 23.11 ± 5.09%, P = 0.0155, Figure 4n), but an increase in average vessel diameter (d21 vs d28: 117.0 ± 5.7 µm vs 126.2 ± 5.3 µm, P = 0.0312, Figure 4o). A significant increase in cross‐sectional vessel area (sham d7 vs severe colitis d28: 731.0 ± 2066 µm^2^ vs 980.8 ± 1617 µm^2^, P < 0.0001, Figure 4p and Figure S9, Supporting Information) observed in CD31‐stained histopathological colon cross sections suggest a dilation of blood vessels and/or an absence of smaller vessels. Furthermore, decreasing normalized blood volume (d21 vs d28: 20.30 ± 3.39 µm^3^ µm^−2^ vs 12.37 ± 3.33 µm^3^ µm^−2^, P = 0.0115, Figure 4q), smaller largest connected component regarding network length (d21 vs d28: 7.64 ± 2.66 mm vs 2.24 ± 0.94 mm, P = 0.0036, Figure 4r) and a decrease in the number of branches (d21 vs d28: 1067 ± 98.9 cm^−2^ vs 807.6 ± 117.3 cm^−2^, P = 0.0111, Figure 4s) indicate a loss of vessels. Subsequently, fractal dimension decreased (d21 vs d28: 1.27 ± 0.48 vs 1.12 ± 0.07, P = 0.0061, Figure 4t), suggesting a less extensive and less intricately interconnected network of vessels as well as a smoother and less convoluted vascular pattern.

## 3 Discussion

In this work, a novel image‐processing pipeline for TAG‐RSOM was developed for label‐free in vivo assessment of changes in the vascular network of murine colitis. Different image segmentation methods were compared and optimized for visualization and quantification of deep vascular patterns revealing decreased network length, vessel coverage, blood volume, and largest component length along with increased average vessel diameter in murine colitis. These novel insights could accelerate the implementation of this technology in preclinical IBD research and translational studies of inflammatory bowel disease.

Given the high sensitivity of optoacoustic imaging for hemoglobin, the large FOV, and the comparatively high penetration depth, TAG‐RSOM holds promise for widespread applications in transmural imaging of the colon in murine models, which could provide a decisive advantage in the research, diagnosis, and monitoring of chronic inflammatory bowel disease. While RSOM has been used for quantification of vascular networks and their oxygenation in subcutaneous xenografts,^[^ ^13^, ^16^ ^]^ in corneal neovascularization^[^ ^17^ ^]^ and in brain imaging of strokes, tumors, and neurodegenerative diseases^[^ ^18^ ^]^, the complexity of the imaging artifacts necessitates adjustment of the image processing protocol to the respective imaging setup and target pathology. Here, we developed and applied an image analysis pipeline tailored for TAG‐RSOM that enables assessment of the vascular network of the murine colon for research on IBD.

For quantification of vascular network changes in a model of chemically‐induced acute colitis, the best results were achieved by a combination of vesselness‐filtering with rule‐based thresholding. Considering the large TAG cross section compared to the murine intestinal lumen, resulting in a uniformly stretched colon wall that minimized vessel overlap, segmentation and characterization of the vascular network were conducted in two dimensions to circumvent the challenge of 3D image segmentation. Moreover, consistent tissue thickness provided a homogeneous light fluence along the imaging FOV, which enabled the use of rule‐based segmentation methods, because intensity variations in the resulting image remained within the same order of magnitude. The use of rule‐based segmentation is advantageous as it avoids the need for meticulously labeled training data,^[^ ^13^ ^]^ yet it can still produce quantitative results that reflect the underlying disease biology.^[^ ^15^, ^19^ ^]^

The presented data analysis pipeline was applied to mice with mild and severe colitis induced through DSS administration. We observed a significant decrease in vessel length, reduced vascular network complexity, decreased vessel coverage, and alterations in other vessel network parameters with increasing inflammation. These changes were confirmed by histological analysis of the cross‐sectional vessel diameter in colon tissue samples and health parameters.^[^ ^12^ ^]^ The increase in RSOM vessel diameter is in line with findings of dilated vessels in the lamina propria and submucosa in DSS‐induced models of colitis^[^ ^20^ ^]^ and has also been described as increased mean vascular density (MVD) in the disease progression in colitis interleukin 10‐deficient mice.^[^ ^21^ ^]^ Furthermore, microvascular endothelial dysfunction^[^ ^22^ ^]^ and increased MVD^[^ ^23^ ^]^ may reflect a key pathophysiological mechanism in human chronic inflammatory bowel diseases.

Previous studies that have measured changes in the vascular network associated with murine colitis required invasive methods such as laparotomy combined with fluorescence microscopy, using fluorescein isothiocyanate‐labeled erythrocytes.^[^ ^24^ ^]^ Other modalities, like multiphoton endomicroscopy, have also been utilized for monitoring vascular changes in vivo, albeit limited to mucosal capillaries due to their inability for transmural imaging.^[^ ^25^ ^]^ Moreover, stereoscopic fluorescence microscopy is promising for imaging the smallest vessels like choroidal microvessels.^[^ ^26^ ^]^

As alternate RSOM enables transabdominal and transmural imaging with a lateral resolution of 40 µm and an axial resolution of 10 µm over a field of view of 12 × 12 × 3 mm with as little burden on animals as possible, which in case of severe colitis are already distressed. Hence, TAG‐RSOM provides a minimally invasive method with low burden and high scientific quality for longitudinal studies where other imaging modalities depend on complex animal welfare considerations and/or are limited to superficial imaging. Notably, analysis of the sham control group revealed minimal impact of this label‐free imaging protocol on the experiment.

Our study is limited by the use of the acute DSS colitis model. While the consistency of disease progression in the DSS model facilitated the establishment of the presented image analysis pipeline, alternative colitis models may offer greater suitability for investigating the pathophysiological changes associated with IBD. Furthermore, future technological improvements in OAI methods with greater spatial resolution might allow the investigation of even smaller microvessels. However, in human IBD, microvessels would already have a diameter in the displayable range (≈150 µm).^[^ ^22^ ^]^ In addition, changes in vascular architecture might be investigated in murine models of colorectal cancers with microvessels with a diameter of around 21 µm, too.^[^ ^27^ ^]^ As with other murine tumors, the effect of vascular‐targeted therapies has been measured with RSOM in these small tumor vessels.^[^ ^16^ ^]^

Moreover, the utilization of multispectral RSOM imaging could enable spectral unmixing, facilitating tissue composition analysis of various endogenous chromophores such as deoxygenated and oxygenated hemoglobin, fat, water, and proteins.^[^ ^28^ ^]^ Compared to deep tissue imaging of the human intestine by tomographic multispectral OAI (MSOT), the utilization of TAG‐RSOM in murine colitis is less affected by depth‐dependent spectral coloring (also known as spectral erosion),^[^ ^29^ ^]^ and could serve as a platform for investigating the theoretical limit of MSOT in IBD.^[^ ^10^ ^]^ TAG‐RSOM in combination with MSOT could thus, offer an excellent experimental setup for further investigations in this domain.

While TAG‐RSOM is introduced as a primarily pre‐clinical research tool, various aspects of this research hold direct implications for clinical care. For instance, Paulus et al. demonstrated the use of orally administered indocyanine green as lumen side contrast agent during MSOT,^[^ ^30^ ^]^ and vessel analysis in the gastrointestinal tract shows promise as OAI systems continue to become miniaturized.^[^ ^31^ ^]^ Hence, our pipeline could inform future quantification efforts in clinical colon imaging. Thus, TAG‐RSOM combined with quantitative vessel analysis presents a new and unique tool for pathophysiologic analysis and pharmaceutical research; it is well suited to improve our understanding of the diagnostic potential of OAI biomarkers in colitis and could in the future be extended to examine vascular changes in Crohn's disease and other inflammatory conditions.

## 4 Experimental Section

Experimental data used in this work were also used to introduce transrectal absorber guide (TAG) RSOM, please see *Buehler* et al. for a more detailed description of the manufacturing of the transrectal absorber guide (TAG) and the imaging protocol.^[^ ^12^ ^]^ To enhance visualization, the resolution of all 2D colon wall projections displayed in Figure 1, 2, 3, 4 has been upscaled by a factor of ten with bilinear interpolation, and segmented colon walls have been upscaled by a factor of ten with a subsequent median filter. The original data used for vessel quantification were available in the supplementary materials.

### Animal Model

All animal procedures were approved by the Animal Welfare and Ethical Review Body at Cancer Research UK Cambridge Institute (project license PE12C2B96, user license I24947753), and issued under the United Kingdom Animals (Scientific Procedures) Act, 1986. Acute colitis was induced in 6‐week‐old BALB/c mice (Charles River, UK) by supplying dextran sulfate sodium (MP Biomedicals, Irvine, CA, USA) dissolved in sterile drinking water ad libitum. A 7‐day cycle of DSS administration followed by two days of drinking water without DSS was used to induce mild colitis, and two 7‐day cycles (3% first cycle, 5% second cycle) were used to induce severe colitis. A sham group underwent all procedures twice while receiving drinking water without DSS with a 7‐day break in the interim.

### TAG‐RSOM Imaging Protocol

Mice were anesthetized using 5% Isoflurane in a gas mixture of 50% oxygen and 50% air. Anesthesia was sustained using a reduced isoflurane concentration between 1% and 2% for a stable respiration rate of 70 to 80 breaths per minute. In vivo imaging of murine colon vasculature was performed using a transrectal absorber guide (TAG) in combination with a commercially available raster scanning optoacoustic mesoscopic (RSOM) imaging system (RSOM Explorer P50, iThera Medical GmbH, Munich, Germany) (Figure S1, Supporting Information). The TAG was placed inside the colon of the mouse to adjust it to the imaging depth of the RSOM and to provide a high contrast surface for exact localization of the intestinal wall. At each time point, two images–one with high‐contrast ink and one with water within the guide–were taken without repositioning of the animal to generate a first localization image followed by a second image with fewer artifacts caused by the TAG.

### Histological Analysis

At the endpoint of each experiment, for each mouse, the colon was dissected and fixed in 10% formalin solution before being embedded into paraffin. Embedding, sectioning, staining with H&E and CD31, and digitization were performed by the CRUK Cambridge Institute Histopathology Core Facility following standard protocols.^[^ ^12^ ^]^ For quantification, the area of venules and arterioles was measured using the software QuPath (version 0.4.3).^[^ ^32^ ^]^

### Reconstruction and Motion Correction

A low‐pass filter (11–33 MHz) was applied to the raw data before the images were reconstructed using a back‐projection algorithm with a voxel size of 20 × 20 × 4 µm^3^, and the motion was corrected for by using software supplied by the manufacturer (viewRSOM Version v2.3.5.2, iThera Medical GmbH, Munich, Germany).

### Preprocessing

Images were processed using Fiji software (ImageJ version 1.53q, National Institutes of Health, USA). To mitigate depth‐dependent periodic reflection artifacts, the volume was filtered in all three dimensions using a 3D Gauss filter. Subsequently, the background was calculated and slice‐wise subtracted using the rolling ball algorithm.^[^ ^33^ ^]^ The colon wall was separated from tissue and TAG, whereby everything but the upper tenth of the colon tube was discarded. To achieve this, average intensity projections of 1 mm segments along the TAG‐axis were calculated for better orientation. The projections were used to mark the highest point of the colon wall and the TAG to estimate the outline of the colon. Individual coordinates for each slice were calculated using linear interpolation. The colon wall was defined as inside standardized circles intersecting these coordinates. Voxels outside these circles were discarded.

All subsequent analysis was performed in two dimensions. For this, the average intensity projection along the z‐axis (depth) of the extracted colon volume was calculated. The sliding paraboloid (Fiji function *Subtract Background*, by Michael Schmid, 2007) method was used instead of the standard rolling ball approach for background subtraction. This choice was made to better preserve elongated structures, such as blood vessels since the paraboloid kernel was less sensitive to significant variations in pixel intensities.^[^ ^34^ ^]^

### Segmentation

Two rule‐based and one learning‐based segmentation methods were used in this study. For the learning‐based approach, data (n = 35 images of N = 10 healthy and diseased mice) from unpublished preliminary experiments were manually annotated and used to train a Random Forest classifier (Ilastik version 1.3.3post3)^[^ ^35^ ^]^ regarding intensity features (Gaussian Smoothing), combined with edge filters (Laplace of Gaussian, Gaussian Gradient Magnitude, Difference of Gaussians) and texture descriptors (Structure Tensor Eigenvalues, Hessian of Gaussian Eigenvalues) on different scales (sigma up to 10). A threshold of 128 was used to binarize the 8‐bit probability maps where the RF was applied.

Moment‐preserving thresholding (referred to as auto threshold (AT)) as well as minimum error threshold on Frangi‐vesselness‐filtered (VF) images at scales from 40 to 100 µm were used as rule‐based segmentation methods.

### Network Quantification and Descriptor Definitions

Before further processing, images were median‐filtered to suppress impulse noise. The segmented vessel images underwent skeletonization (Fiji function *skeletonize 2D/3D*), and the edges of the images were eroded by 200 µm to encounter edge artifacts caused by segmentation and skeletonization before subsequent analysis (Fiji function *Analyze Skeleton 2D/3D*). For this, the resulting FOV used for the analysis area was ± 18° relative to the apex of the cylindrical TAG. Differences in the placement of the TAG across individual mice led to variations in the colon segment length available for analysis. Consequently, the network descriptors network length, vessel coverage, number of branches, and blood volume were normalized to the specific analysis area employed. The fractal dimension was calculated by box counting (Fiji function *Fractal Box Count*), employing box sizes up to 64. For a more detailed description of the network descriptors see Table 1.

### Statistics

Each group was first tested for Gaussian distribution using the Shapiro–Wilk normality test. For comparison of the sham control group with the mild colitis group, and for histological analysis, ordinary one‐way ANOVA was used for normally distributed, and Kruskal–Wallis test in case of non‐normally distributed, data. Multiple comparisons were corrected by using Šidák's and Dunn's tests, respectively. For the repeated measurements of the severe colitis group and the comparison of segmentation methods, one‐way ANOVA with Geisser–Greenhouse correction was used for normally distributed, and the Friedman test in the case of non‐normally distributed, data. Tukey hypothesis testing was used to correct for multiple comparisons tests on normal distributions and Dunn's test on non‐normally distributed data. The Mann–Whitney test was employed to compare the cross‐sectional vessel diameter in CD31‐stained histological colon sections between the mild/severe colitis and sham groups. GraphPad Prism software (Version 9, GraphPad Software, Inc., San Diego, CA, USA) was used for all analyses. Two‐tailed P‐values < 0.05 were considered to be statistically significant.

## Conflict of Interest

M.J.W., F.K., and A.P.R. are shared patent holders with iThera Medical GmbH (Munich, Germany) on an optoacoustic imaging system/software, however, different from the one described in the study.

## Author Contributions

S.E.B. and A.P.R. contributed equally to this work. A.B., F.K., S.E.B., and A.P.R. designed the study. A.B. developed the T.A.G. A.B. and E.L.B. performed all animal experiments. A.B. performed all imaging analyses. M.E. scored histopathology. F.K. scored endoscopy. A.B., F.K., S.E.B., and A.P.R. analyzed the data. A.B., E.L.B., E.N., M.E., P.L., F.W., H.M., R.R., M.E.O., L.P.P., U.R., O.F., M.F.N., J.W., M.J.W., F.K., S.E.B., and A.P.R. interpreted the data. A.B. wrote the first draft of the manuscript. The manuscript was critically reviewed by all authors.

## Supporting information

Supporting Information

## Data Availability

The data that support the findings of this study are available from the corresponding author upon reasonable request. Code for image segmentation, vessel quantification, and exemplar TAG‐RSOM data are available on GitHub (https://github.com/AdrianBuehler/TAG‐RSOM‐Vessel‐Analysis). All imaging data is openly available: https://doi.org/10.17863/CAM.110936
