# Supplementary material for: In Vivo Assessment of Deep Vascular Patterns in Murine Colitis Using Optoacoustic Mesoscopic Imaging
Source: Adv Sci (Weinh). 2024 Oct 22;11(45):2404618. doi: 10.1002/advs.202404618 (PMC11615813; doi:10.1002/advs.202404618)
Supplement: Supplementary file 1 — Supporting Information [file ADVS-11-2404618-s001.docx]

Supporting Information

***In vivo* assessment of deep vascular patterns in murine colitis using optoacoustic mesoscopic imaging**

Adrian Buehler, Emma Brown, Emmanuel Nedoschill, Markus Eckstein, Petra Ludwig, Felix Wachter, Henriette Mandelbaum, Roman Raming, Mariam-Eleni Oraiopoulou, Lars-Philip Paulus, Ulrich Rother, Oliver Friedrich, Markus F. Neurath, Joachim Woelfle, Maximilian J. Waldner, Ferdinand Knieling, Sarah E. Bohndiek*†, Adrian P. Regensburger*†


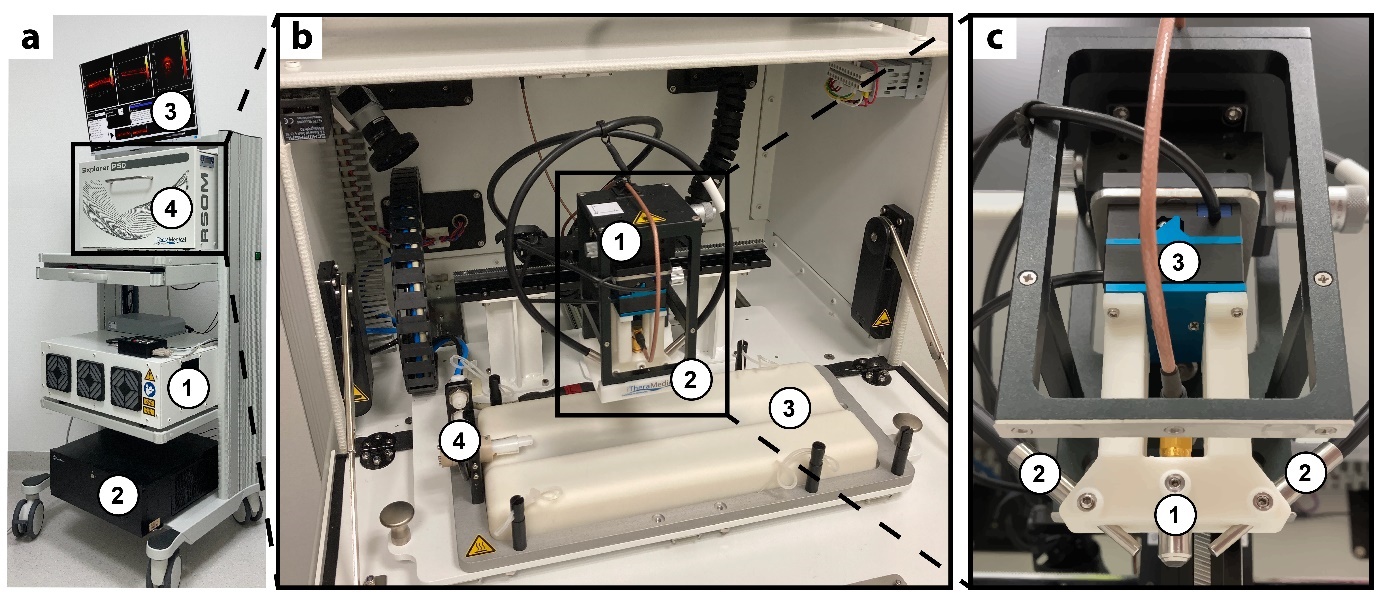
**Figure S1.** Optoacoustic imaging system

**a** A commercially available raster-scanning optoacoustic mesoscopic (RSOM) imaging system (RSOM Explorer P50, iThera Medical GmbH, Munich, Germany) was used in this study. The main components include a 532 nm laser (1), a processing unit (2), a monitor (3), and an optically isolated imaging chamber (4). **b** The chamber contains a scan head (1) with a mounted water bath (2) that can be translated along all three spatial axes. During imaging, the animal is placed on a warming pad (3), and anesthesia is supplied via a nose cone (4). **c** Inside the scan head, the single ultrasound transducer (1) and the two light cables (2) are mounted on an XY-stage (3) to raster over an area of up to 12 x 12 mm^2^.

**
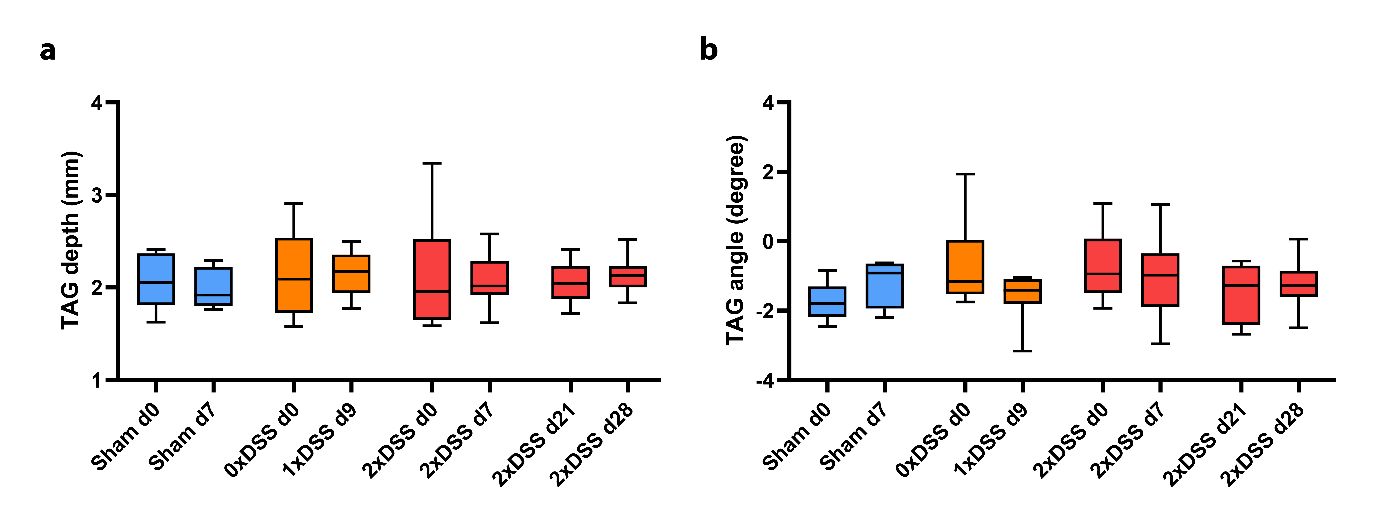
**

**Figure S2.** Transrectal absorber guide placement during raster-scanning optoacoustic mesoscopy.

**a** The transrectal absorber guide (TAG) was placed at a consistent distance from the scan head with no significant deviations between groups. **b** The angle relative to the XY-plane did not deviate significantly between time points. Whiskers correspond to minimum and maximum value.


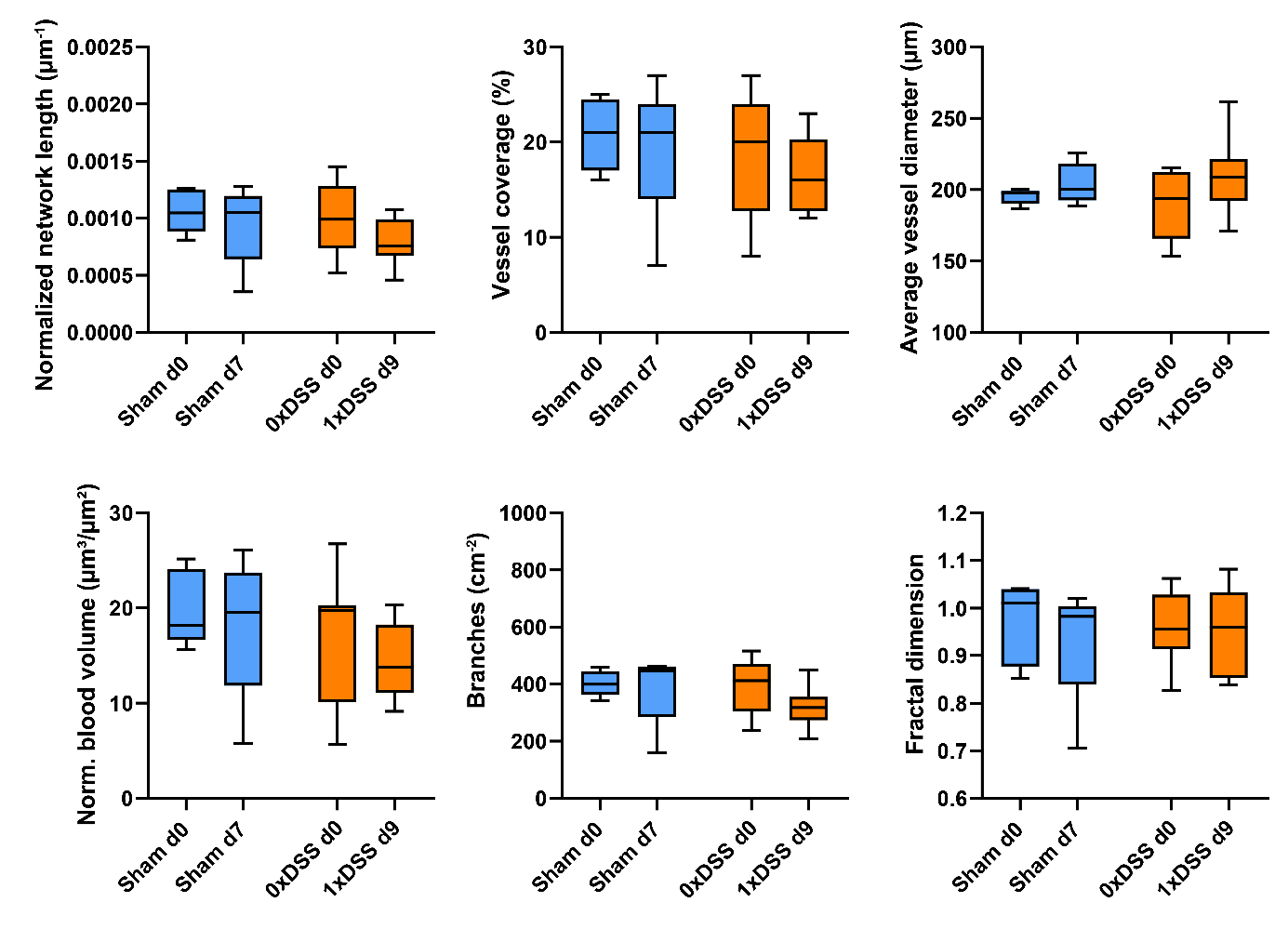


**Figure S3.** Quantification of mild colitis using auto threshold.

Statistical and topological parameters calculated based on TAG-RSOM using auto threshold as segmentation method only. Sham group N = 5 (blue), mild colitis N = 10 (orange), whiskers correspond to minimum and maximum value.


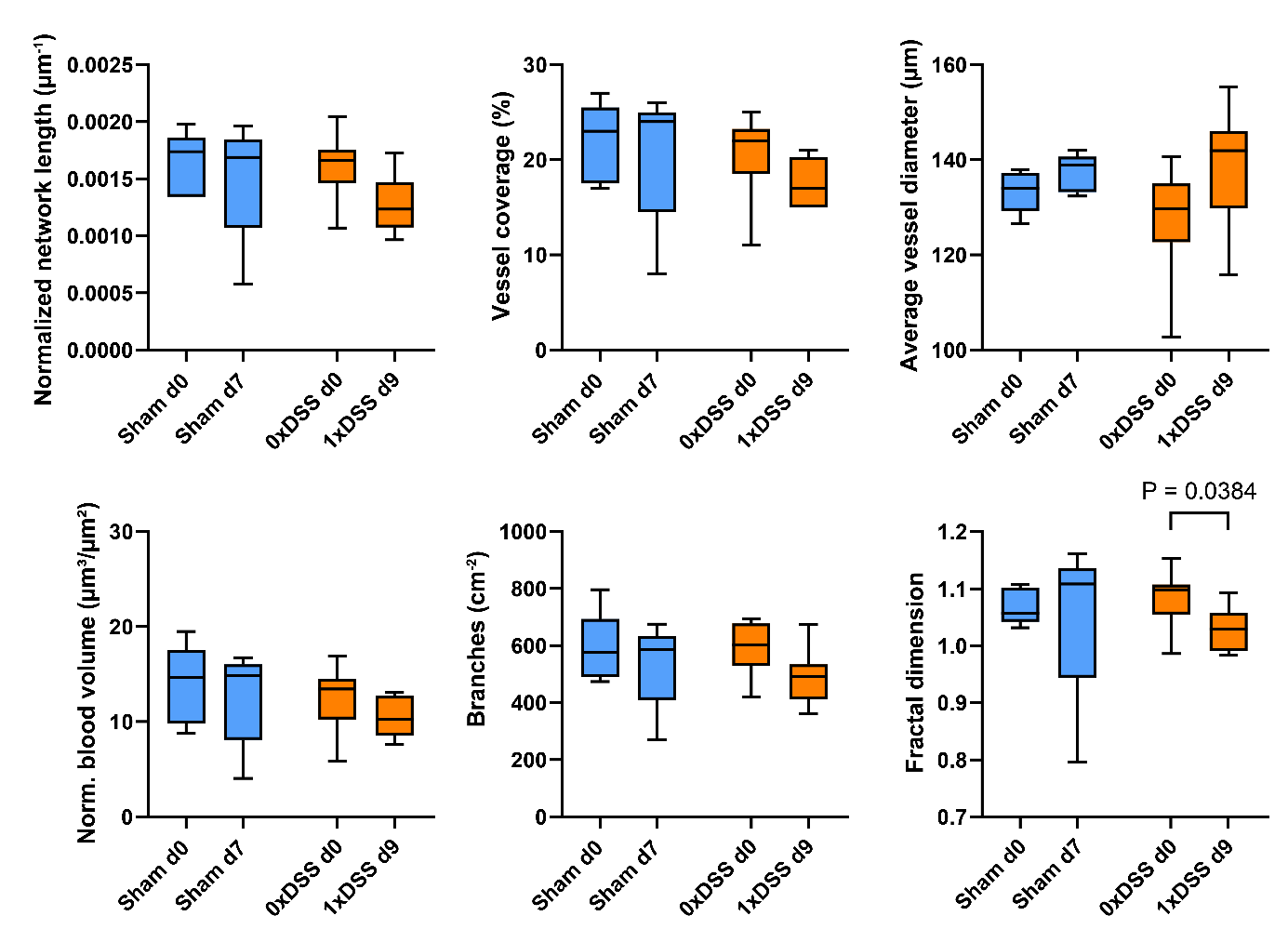


**Figure S4.** Quantification of mild colitis using Random Forest.

Statistical and topological parameters calculated based on TAG-RSOM using Random Forest as segmentation method. Sham group N = 5 (blue), mild colitis N = 10 (orange), whiskers correspond to minimum and maximum value.


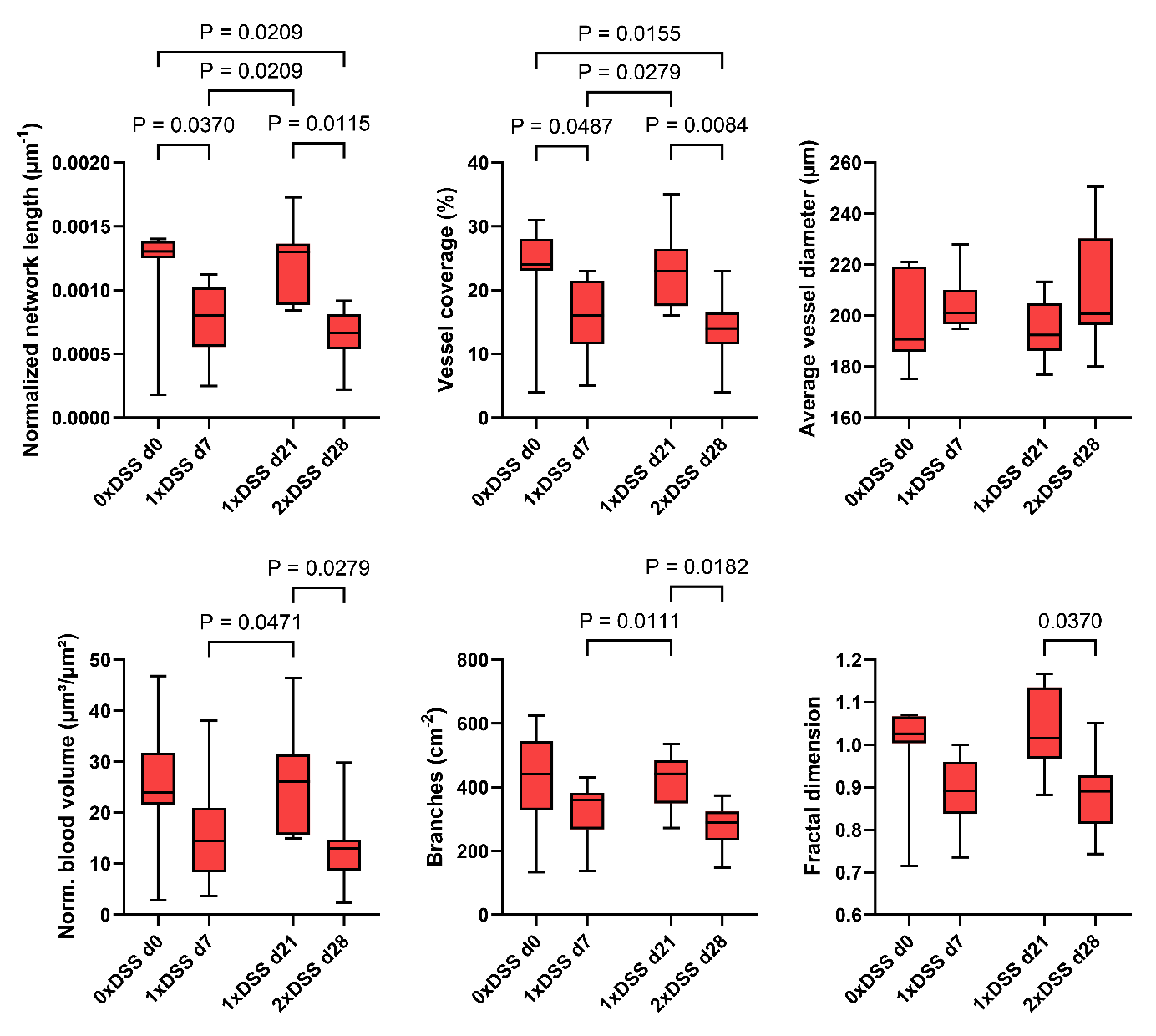


**Figure S5.** Quantification of severe colitis using auto threshold.

Statistical and topological parameters calculated based on TAG-RSOM using auto threshold as segmentation method only. Severe colitis N = 9 (red), whiskers correspond to minimum and maximum value.


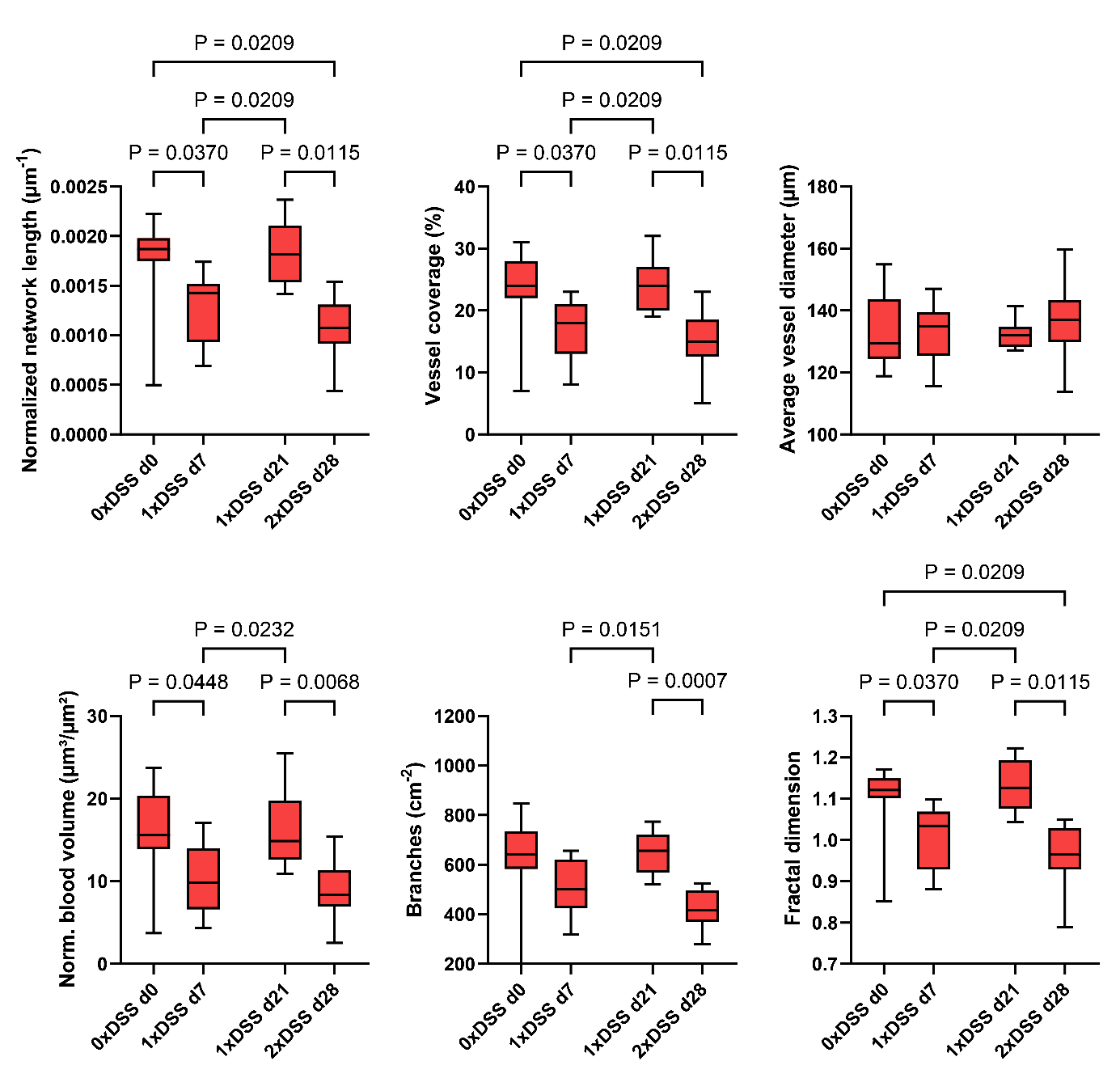


**Figure S6.** Quantification of severe colitis using Random Forest.

Statistical and topological parameters calculated based on TAG-RSOM using Random Forest as segmentation method. Severe colitis N = 9 (red), whiskers correspond to minimum and maximum value.


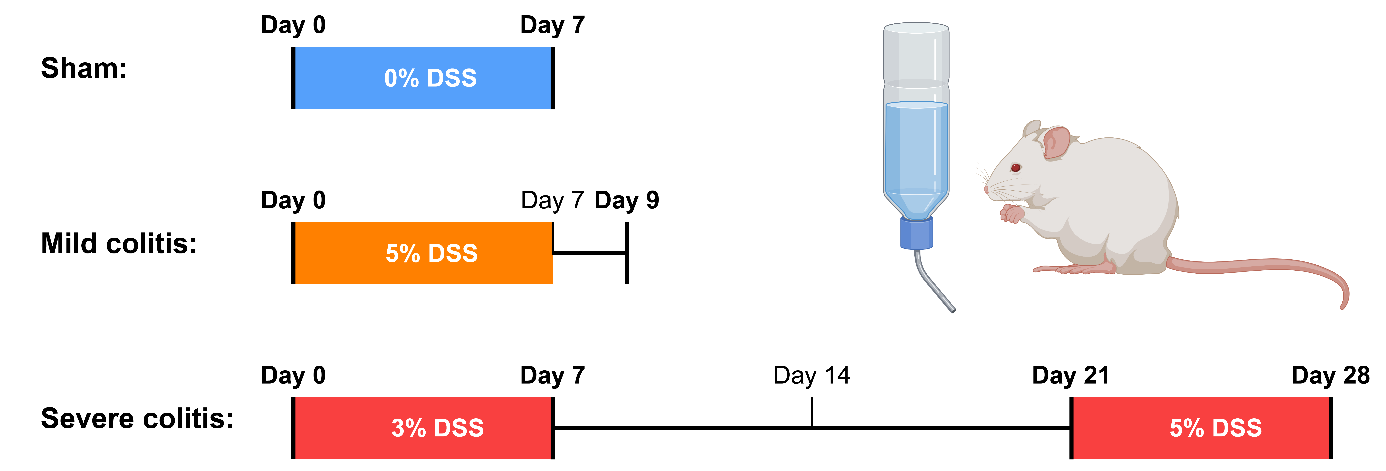


**Figure S7.** Study groups for validating the image-processing pipeline for TAG-RSOM

A sham control group (N = 5) was used to assess the impact of the imaging protocol on the animals. Mild colitis was induced by 7-day-cycle of 5% DSS, followed by two days of drinking water. Severe colitis was induced by two cycles of DSS administration (3% in the first cycle, 5% in the second cycle), with a two-week healing period in between. TAG-RSOM imaging and colonoscopy were performed on days marked in bold. Histology and *ex vivo* colon length measurements were conducted at the endpoint of each group.


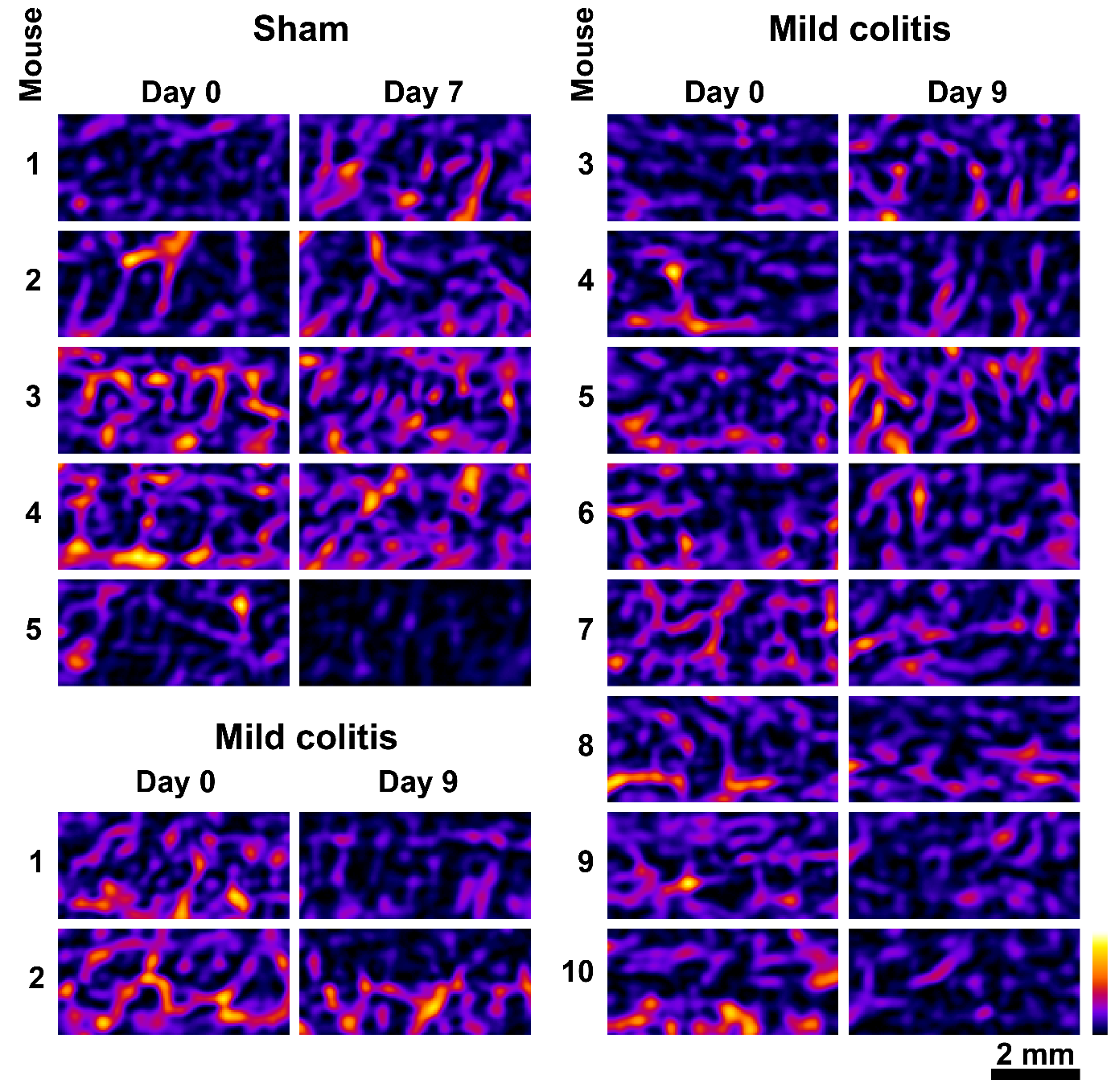


**Figure S8.** RSOM imaging of sham and mild colitis group

This figure shows RSOM imaging data of the sham and mild colitis groups after preprocessing. Mild signs of inflammation were observed on day 9 in the mild colitis group. For each subject and imaging time point, a representative section (5,2 x 2,4 mm^2^) of the analysis area was selected to account for variations in the size and shape of the analysis areas. The complete dataset is openly available.


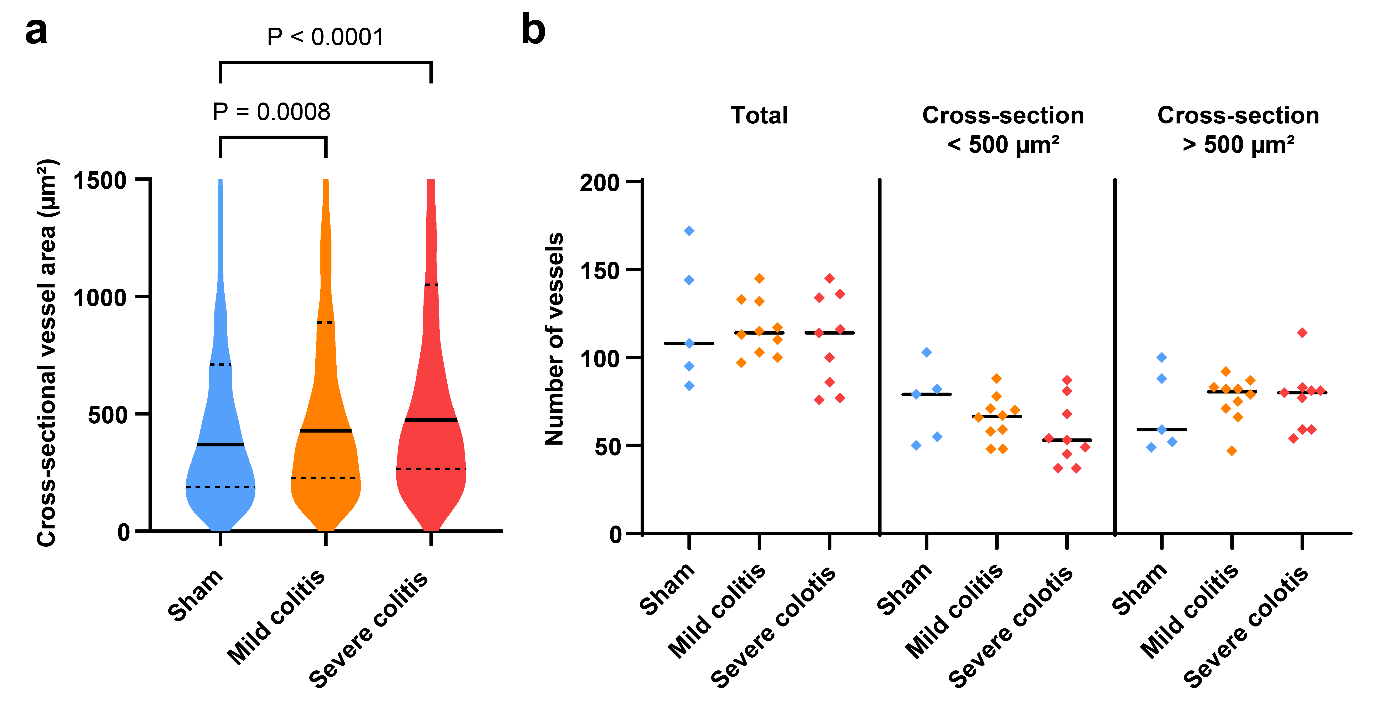


**Figure S9.** Vessel analysis on CD31-stained histopathological colon cross-sections.

**a** The cross-sectional vessel area increases with colitis severity (total number of vessels: shame = 597; mild colitis = 1165; severe colitis = 984). Cross-sectional areas greater than 1500 µm² are not shown. Kruskal-Wallis test was used to compare ranks. **b** Increase in the proportion of large vessels represented by the number of vessels larger or smaller than 500 µm². The number corresponds to vessels found on a single histological colon cross-section.


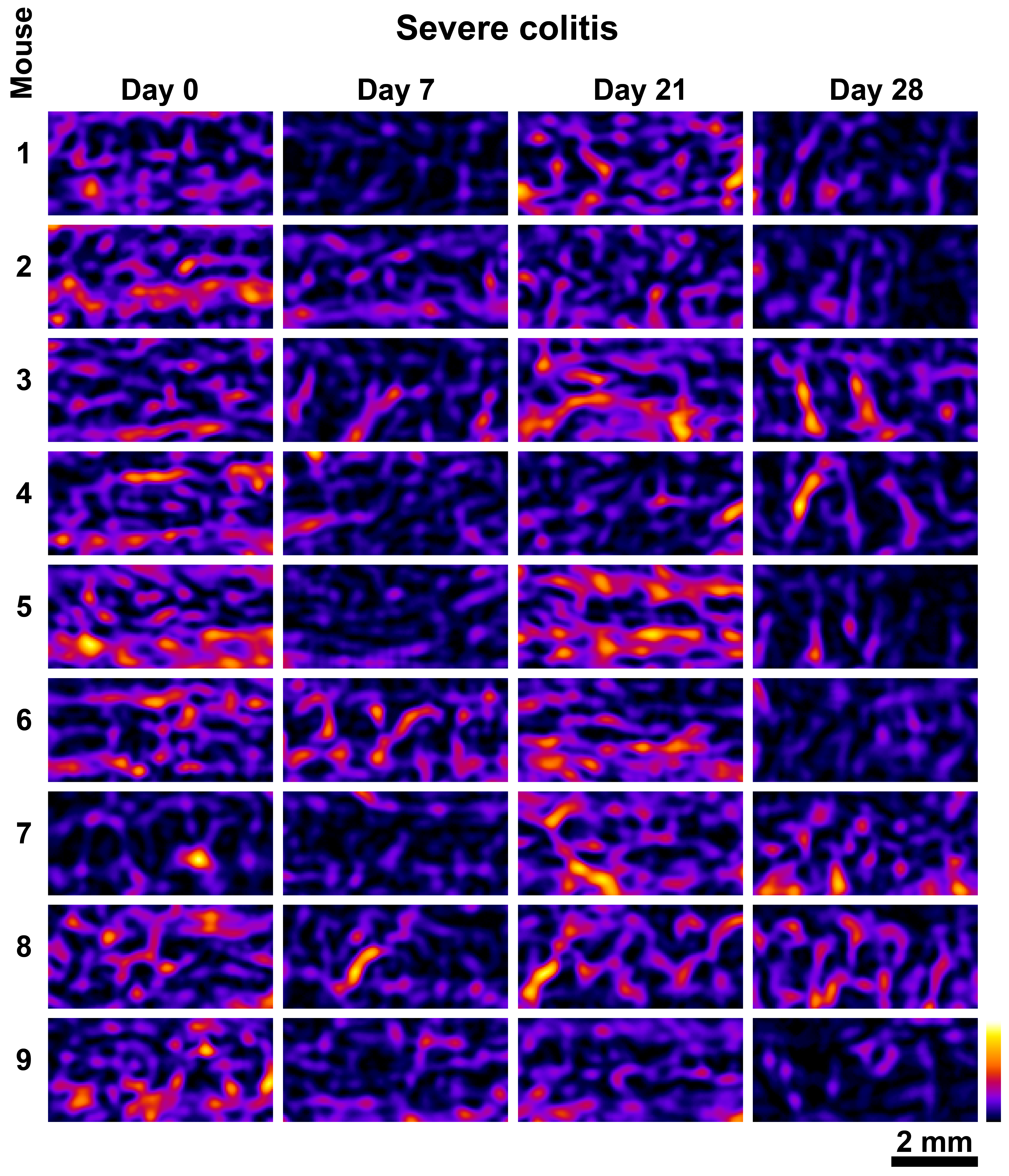


**Figure S10.** RSOM imaging of severe colitis

This figure shows RSOM imaging data of the severe colitis group after preprocessing. Mild signs of inflammation were observed on day 7 and severe inflammation was observed on day 28. For each subject and imaging time point, a representative section (5,2 x 2,4 mm^2^) of the analysis area was selected to account for variations in the size and shape of the analysis areas. The complete dataset is openly available.
